# Relationship between the Antifungal Activity of Chitosan–Capsaicin Nanoparticles and the Oxidative Stress Response on *Aspergillus parasiticus*

**DOI:** 10.3390/polym14142774

**Published:** 2022-07-06

**Authors:** Cynthia Nazareth Hernández-Téllez, Ana Guadalupe Luque-Alcaraz, Sahily Alejandra Núñez-Mexía, Mario Onofre Cortez-Rocha, Jaime Lizardi-Mendoza, Ema Carina Rosas-Burgos, Aarón de Jesús Rosas-Durazo, Norma Violeta Parra-Vergara, Maribel Plascencia-Jatomea

**Affiliations:** 1 Departamento de Ingeniería Biomédica, Universidad Estatal de Sonora, Ley Federal del Trabajo S/N, Col. Apolo, Hermosillo 83100, Sonora, Mexico; cynthia.hernandez@ues.mx (C.N.H.-T.); aaron.rosas@ues.mx (A.d.J.R.-D.); 2 Microbiology and Mycotoxins Laboratory, Departamento de Investigación y Posgrado en Alimentos, Universidad de Sonora, Blvd. Luis Encinas y Rosales S/N, Col. Centro, Hermosillo 83000, Sonora, Mexico; sahilynunez@outlook.es (S.A.N.-M.); mario.cortez@unison.mx (M.O.C.-R.); carina.rosas@unison.mx (E.C.R.-B.); violeta.parra@unison.mx (N.V.P.-V.); 3 Biopolymer Laboratory, Centro de Investigación y Desarrollo en Alimentación, A.C., Carretera Gustavo Enrique Astiazarán Rosas, No. 46, Hermosillo 83304, Sonora, Mexico; jalim@ciad.mx

**Keywords:** acute toxicity, nanomaterials, biopolymer, bioactive compounds, phytopathogenic fungi

## Abstract

The fungus *Aspergillus parasiticus* is a contaminant in agricultural crops and its eradication involves the indiscriminate use of harmful synthetic pesticides. In the search for antifungal agents of natural origin, chitosan (Q) and capsaicin (C) are coupled in the form of nanoparticles (Np), which can possess a direct application under specific conditions. Due to their small size, Np can cross through the cell wall, taking the cells into a pro-oxidant environment known as “oxidative stress”, which presents when the reactive oxygen species (ROS) surpass the number of antioxidants in the cell. In the present investigation, nanoparticles of chitosan (Np Q) and nanoparticles of chitosan-capsaicin (Np QC) with an average diameter of 44.8 ± 20.6 nm and 111.1 ± 14.1 nm, respectively, were synthesized, and there was a zeta potential of + 25.6 ± 0.7 mV and + 26.8 ± 6.1 mV, respectively. The effect of the concentration of Np Q (A, B, C, and D), of Np QC (A, B, C, and D), and capsaicin in a solution (control) was evaluated on the viability of the spores, the accumulation of intracellular ROS, and the morphometric changes of *A. parasiticus*. Acute toxicity of the Np was determined utilizing bioassays with *Artemia salina*, and acute phytotoxicity was evaluated in lettuce seeds (*Lactuca sativa*). According to ROS results, capsaicin (control) did not induce oxidative stress in the cell; otherwise, it was observed to have an elevated (*p* < 0.05) accumulation of ROS when the concentration of Np Q increased. For both, Np Q and Np QC, an inverse physiological pattern relating spore viability and ROS accumulation in the fungus was found; the viability of spores decreased as the ROS accumulation increased. The spore viability of *A. parasiticus* diminished upon increasing the concentration of chitosan (0.3–0.4 mg/mL) in the Np, while the intracellular accumulation of ROS increased proportionally to the concentration of the nanomaterials in the treatments of Np Q and Np QC. On the other hand, Np QC presented a lower (*p* < 0.05) toxicological effect in comparison with Np Q, which indicates that the incorporation of bioactive compounds, such as capsaicin, into nanoparticles of chitosan is a strategy that permits the reduction of the toxicity associated with nanostructured materials.

## 1. Introduction

The typical piquant flavor of the fruits of the genus *Capsicum* is due to the presence of a group of compounds known as capsaicinoids, which synthesize and accumulate in the placental tissue. Among these, capsaicin is an alkaloid with the formula C1_8_H_27_O_3_N (trans-8-methyl-N-vanillyl-6-nonenamide) that is solid at room temperature, with a molecular weight of 305.4 Da. It is soluble in ethanol, acetate, and fatty acids, and practically insoluble in cold water [1]. The quantification of capsaicin in chili peppers and capsaicine loaded in nanoparticles is made possible by high pressure liquid chromathography (HPLC) [2,3,4]. In a recent study, the authors combined HPLC direct analysis in real time (DART) with the time of flight-mass spectrometry (TOF-MS) method for quantifying capsaicin in 46 types of chili peppers of three different varieties (*Capsicum annuum* L., *C. baccatum* L., and *C. frutescens* L.) [5], obtaining novel results in terms of the correlation between the two methods of capsaicin quantification and, in the case of Biquinho (*C. chinense*), the amount of seeds in the fruits. With respect to biological characterization, it is known that capsaicin possesses antimicrobial properties, and there is evidence of its effect on fungal species, such as *Aspergillus flavus* [6], *Sphaeropsis sapinea*, *Leptographium procerum* [7], *Botrytis cinerea*, and *A. niger* [8]. Recently, the possibility of increasing the bioavailability and antimicrobial action of capsaicin upon coupling it with natural polymers, such as chitosan, has been reported, with the purpose of forming chitosan–capsaicin-based nanometric systems with antimicrobial properties [9,10]. In this respect, utilizing disk diffusion tests, it has been demonstrated that an increase in the amount of capsaicin in chitosan films gradually improves activity against bacteria, such as *Escherichia coli*, *Salmonella enterica*, *Proteus mirabilis*, *P. vulgaris*, *Pseudomonas aeruginosa*, *Enterobacter aerogenes*, *Staphylococcus aureus*, *Streptococcus mutans*, and *Bacillus thuringiensis*, as well as anti-quorum sensing activity against *Chromobacterium violaceum* (CV026 and ATCC 12472). It is important to mention that the authors of that study employed film-former solutions instead of material in the form of film, which could have exerted an effect on the material’s bioactivity [11]. However, care must be taken to ensure the purity of chitosan, as protein, metal, or other contaminants could potentially cause many deleterious effects, both in derivative syntheses and in dosage forms [12]. Zebrafish embryo exposure to chitosan nanoparticles (200 nm) caused malformations and resulted in a decreased hatching rate, increased the rate of cell death, and caused high expression of reactive oxygen species, which was concentration-dependent [13,14].

Interest in the study of chitosan-based microsystems and nanosystems has increased due to potential applications in the encapsulation of bioactive compounds, which have been associated with biological properties, such as biodegradability, biocompatibility, and low or null toxicity, as well as for a capacity to form films, membranes, gels, pellets, fibers, and micro- and nanoparticles [15] For example, Tran and Hadinoto [16] synthesized nanocapsules of chitosan–capsaicin, which presented a prolonged release to the bioactive compound, rendering this molecule an attractive vehicle for the oral bioavailability of capsaicin. There is evidence of the capacity of capsaicin to act as an antioxidant agent on the incorporation of the biomolecule into chitosan-based films, as the OH groups of the molecular structure can induce an antioxidant effect via molecular interaction [11]. In addition, an antimicrobial effect of polyethylene glycol–capsaicin films has been reported in *E. coli* [17], as well as in the apoptosis of cancerous cells through the intracellular elevation of reactive oxygen species (ROS) [1,18]. 

With respect to the antimicrobial activity of nanoparticle materials, previous studies using fluorescence microscopy analysis revealed affectations in membrane integrity, cell-wall damage, and the induction of oxidative stress, mainly at the early growth stages of *A. niger* exposed to chitosan nanoparticles loaded with pyrrole-2-carboxylic acid [19]. In a similar study, utilizing low cytometry, there was evidence that metallic nanoparticles and metallic oxides (Cu, Zn, CuO, and ZnO) are capable of penetrating the bacteria and inducing an excessive generation of ROS. In addition, an increase in membrane permeability was observed, which could be associated with affectations in antibiotic-resistant genes during the proliferation of bacterial species present in samples of leachate from the Shanghai Laogang landfill [20]. Similar results have been reported for Ag-, CuO-, and ZnO- based nanoparticles/ions, whose concentrations in the environment are capable of promoting the natural transformation of plasmids that codify for the synthesis of antibiotic-resistant genes in *Acinetobacter baylyi*, *E. coli*, and a mixed culture of aquatic microbiota [21]. These have also been associated with the over-production of ROS and damage to the cellular membrane [22] and chitosan’s ability to destabilize the permeability of the outer membrane of bacterial cells [23].

The term ROS is used to describe all intermediate oxygen reagents that include oxyradicals (singlets and doublets) and non-radicals (H_2_O_2_). Oxyradicals are harmful when they are produced in large amounts, because they can indiscriminately react with biomolecules. The accumulation of ROS within the cells and their release into the culture medium can be analyzed qualitatively by means of an in vitro cell culture model in order to understand the mechanism of cell death. Highly reactive species are continually generated during aerobic metabolism, and an excess of these compounds can give rise to oxidation, finally occasioning cell death [24]. 

The objective of this work was to synthesize and characterize chitosan–capsaicin nanoparticles and to evaluate the biological properties that lead to the elucidation of the possible induction of ROS and antifungal activity against *A. parasiticus*, a toxigenic fungus of importance in foods.

## 2. Materials and Methods

### 2.1. Materials

For the preparation of the nanoparticles, medium-molecular-weight commercial chitosan was utilized (448877; Sigma-Aldrich, St. Louis, MO, USA). As a control, commercial capsaicin was employed (360376; Sigma-Aldrich, St. Louis, MO, USA).

### 2.2. Culture and Propagation of the Fungus

The *A. parasiticus* (ATCC 16992) strain was inoculated in an Erlenmeyer flask containing potato dextrose agar (BD Bioxon, Estado de Mexico, Mexico) acidified with tartaric acid at 10% (*w*/*v*), incubating this mixture for 5 days at 25 °C. For preparation of the inoculum, the fungus culture and the spores were resuspended in an Erlenmeyer flask by means of the addition of sterile Tween 80 solution (0.01% *v*/*v*), with shaking for 1 min with the aid of a magnetic shaker. After this, to determine the concentration of the spores in the inoculum, a drop of the suspension was placed in a Neubauer chamber for direct count under the microscope. For the bioassays, the inoculum was adjusted to a concentration of 4 × 10^6^ spores/mL [15].

### 2.3. Preparation of the Nanoparticles

The nanoparticles were obtained through the nanoprecipitation method, according to the procedure described by Luque-Alcaraz et al. (2012) [25]. A solution of chitosan at 0.4% (*w*/*v*) in 100 mL of acetic acid at 1% (*v*/*v*) was prepared. From this solution, 1 mL from the solvent phase was added drop-by-drop to the non-solvent phase (25 mL of methanol, containing or not containing capsaicin at a concentration of 0.20 mg/mL), maintaining the solution under magnetic shaking (500 rpm). The drip was maintained under a constant flow of 0.870 mL/min, utilizing a peristaltic pump (Gilson Minipuls 3, Villiers-le-Bel, France). Chitosan nanoparticles without capsaicin were used as control.

### 2.4. Preparation of the Capsaicin Solution (Control)

A total of 4.5 mg of capsaicin was dissolved in a methanol solution containing 125 µL of pure methanol and 375 µL of distilled water. The concentrations utilized are shown in Table 1. 

### 2.5. Physical and Physicochemical Characterization of Nanoparticles

#### 2.5.1. Form, Size, and Superficial Load

The form and size of the nanoparticles were determined by means of an atomic force microscope in Veeco Bruker equipment (Terminal Drive, Plainview, NY, USA). The images were obtained in tapping mode at a scan range of 3 µm at a scan rate of 0.5 Hz. 

The diameter and the zeta potential were determined by dynamic light dispersion analysis (Möbiuz; Wyatt Technology, Santa Barbara, CA, USA). A total of 90 µL was placed in a cell of the instrument, which was equipped with vertically polarized laser with a 488 nm wavelength (2W). The detection angle was maintained at 90° with respect to the incident light beam, and the averages of three replications were obtained, each with a duration of 60 s, at room temperature [26].

#### 2.5.2. FT-IR and Nanoparticle Entrapment Capacity

The interaction between chitosan and capsaicin was analyzed by Fourier Transformed Infrared Spectroscopy (FT-IR). The spectra were obtained in a Perkin–Elmer FT-IR Spectrum GX (Shelton, CT, USA) with an average of 16 scans within a spectral range of 4000 to 400 cm^−1^. The analysis was carried out in liquid media. The identification and content of the capsaicinoids in the samples were determined by comparing the retention times of the capsaicin standard (Sigma-Aldrich Co., St. Louis, MO, USA): The experiment was carried out in an Agilent Technologies 1260 Infinity High Performance Liquid Chromatograph equipped with a photodiode detector, a Zorbax Eclipse XDB-C18 semi-preparative column (5 µm particle size, 250 × 9.4 mm i.d.), and a 20 µL injection loop. Column temperature was set at 20 °C. Elution of components was carried out at a flow rate of 2 mL/min using a linear gradient from 50:50 to 0:100 during the first 9 min [27].

### 2.6. Toxicological Analysis of the Nanoparticles

#### 2.6.1. Acute Toxicity in *Artemia salina* Nauplii

The shrimp-brine nauplii of *A. salina* were incubated during 24 h for hatching in sterile seawater, at room temperature, with controlled aeration and artificial illumination [28]. After hatching, the nauplii of *A. salina* were separated into groups of 10 to 15 specimens, transferring them with a Pasteur pipette into assay tubes of 18 × 150 mm containing sterile seawater, to which different concentrations of Np Q and Np QC of capsaicin (control) had been previously added (Table 1). The final volume was increased to 5 mL with sterile seawater, and all of the tubes were maintained at room temperature under controlled illumination. After 24 h, the surviving nauplii were counted with the help of a magnifying glass, and the percentage of mortality of the quantity of live organisms counted among the controls was determined. Once the test was concluded, the nauplii were sacrificed through the addition of 100 mL of a solution of phenol at 5% (*w*/*v*) [29].

#### 2.6.2. Acute Phytotoxicity in Lettuce Seeds (*Lactuca sativa*)

The percentage of germination of the seeds and the elongation of the root were measured in distilled water for the control treatment. Each of the treatments (Table 1) was evaluated in triplicate [30,31]. Briefly, a piece of filter paper was placed in each Petri dish, and 5 mL of the nanoparticle suspension was added, with different concentrations. Above the humidified filter paper, 20 seeds were placed in each box in equidistant fashion. The boxes were left to incubate in a germinator, at a relative humidity of 99%, at 25 °C, for 5 days. After this, the seeds that germinated in each plate were counted. The effect of the treatments on the seeds was calculated according to the equations described below.

Relative seed germination (RSG) (%), Equation (1):

(1) $$\mathrm{RSG}\;\left(\%\right)=\frac{\mathrm{Seeds}\;\mathrm{germinated}\;\mathrm{with}\;\mathrm{treatment}}{\mathrm{Seeds}\;\mathrm{germinated}\;\mathrm{with}\;\mathrm{control}}\times 100$$

Relative radicle elongation (RRE) (%), Equation (2):

(2) $$\mathrm{RRE}\;\left(\%\right)=\frac{\mathrm{Radicle}\;\mathrm{elongation}\;\mathrm{with}\;\mathrm{treatment}}{\mathrm{Radicle}\;\mathrm{elongation}\;\mathrm{with}\;\mathrm{control}}\times 100$$

Germination index (GI) (%), Equation (3):

(3) $$\mathrm{GI}\left(\%\right)=\frac{\mathrm{RSG}\;\times \;\mathrm{REE}}{100}$$

Normalized residual percentage of germinated seeds (IGN) (%), Equation (4):

(4) $$\mathrm{IGN}\;\left(\%\right)=\frac{{\mathrm{Germ}}_{\mathrm{treatment}}-{\mathrm{Germ}}_{\mathrm{control}}}{{\mathrm{Germ}}_{\mathrm{control}}}$$

Normalized residual percentage elongation of the root of the germinated seeds (IER) (%), Equation (5):

(5) $$\mathrm{IER}\;\left(\%\right)=\frac{{\mathrm{Elongation}\;}_{\mathrm{treatment}}-{\mathrm{Elongation}}_{\mathrm{control}}}{{\mathrm{Elogation}}_{\mathrm{control}}}$$

### 2.7. Antifungal Activity of the Nanoparticles of A. parasiticus

#### 2.7.1. Morphometric Analysis 

To evaluate the effect of the nanoparticles on the average diameter of the *A. parasiticus* spores, an image-processing analysis was utilized. One hundred mL of the spore suspension of the fungus was placed in a sterile 96-well microplate and incubated for 4 h at 27 ± 2 °C (EI60 Novatech, San Pedro Tlaquepaque, Jalisco, Mexico). Subsequently, 100 µL of the nanoparticle suspension was added to each well at different concentrations, again incubating the microplates for 4 h at 27 ± 2 °C. Next, utilizing a micropipette, random samples of the fungus developed in the wells were obtained and deposited on a slide for observation. The average spore diameter was determined via image analysis using Imagen-Pro Plus version 6.3 software (2008) (Media Cybernetics, Inc., Bethesda, MD, USA), employing an optic microscope (Olympus CX311RTSF, Tokyo, Japan) connected to an Infinity 1 camera (Lumenera Corp., Ottawa, ON, Canada) with the 40X objective [32].

#### 2.7.2. Spore Viability

Quantification of the relation of the number of viable spores utilizing the XTT salt (Sigma-Aldrich, St. Louis, MO, USA) (2,3-bis (2-methoxy-4-nitro-5-2H-tetrazolium hydroxide) was carried out using a colorimetric assay in the presence of an electron-coupling agent, menadione (Sigma-Aldrich, St. Louis, MO, USA) [33]. A solution of XTT at a concentration of 2 mg/mL of saline solution was prepared (Pisa, Guadalajara, Jalisco, Mexico) and passed through a filter with a 0.2 µm pore size (PALL Corporation, Port Washington, New York, NY, USA). A solution of menadione in acetate (J.T. Baker, Center Valley, PA, USA) was prepared at a concentration of 10 mM and added to a concentration solution of 1.0 mM, using an aliquot of 1 mL and homogenizing it with 10 mL of physiological saline solution. A total of 100 µL of the spore solution (4 × 10^6^ spores/mL) was deposited in the small wells of the microplate and incubated for 4 h in an incubator (EI60 Novatech, San Pedro Tlaquepaque, Jalisco, Mexico) at 27 ± 2 °C. Later, the treatments were added (Table 1), depositing 100 µL of each of the treatments into each of the respective wells. After applying the treatments and at the end of the incubation time, 50 µL of the XTT solution and 7 µL of the menadione solution were added into each small well of the microplate, and this was incubated again for 3 h [29]. Finally, the absorbance of each microplate was read in a spectrophotometer (ThermoScientific, MultiskanGo, Thermo Fisher Scientific Oy, Osakeyhtiö, Finland), at a wavelength of 450 nm.

#### 2.7.3. Quantification of Reactive Oxygen Species (ROS) 

To quantify the production of ROS induced by the exposure of the nanoparticles, we used a method with dichlorodihydrofluorescein diacetate (DCFH-DA) [34]. A microplate fluorescence reader was employed to evaluate the production of ROS induced by the treatment at different concentrations. This method is based on the diffusion of DCFDA. Within the fungal cell, the DCFDA is deacetylated by esterase cells into a non-fluorescent compound that later is oxidized by the ROS to 2′7′-dichlorofluorescein, which emits fluorescence when it is excited. The DCFA-DA method has been utilized to determine the production of ROS in different types of cells. The DCFH-DA is an indicator of intracellular oxidative stress and allows for the quantification of certain produced ROS. Briefly, 100 µL (4 × 10^6^ spores/mL) of the *A. parasiticus* spore suspension was added to each well in a 96-well microplate. The microplate was incubated for 4 h at 27 ± 2 °C (EI60 Novatech, San Pedro Tlaquepaque, Jalisco, Mexico) and the nanoparticles were then added following the procedure described to evaluate the effect of nanomaterials on viability. In the same manner, another microplate was used for the controls of the spores and the water for the calculation of ROS production. After adding the nanoparticles to the different concentrations, the plates were incubated again for 24 h at the same temperature. Then, with the aid of a micropipette, the culture medium was removed, 50 µL of the DCFH-DA solution was added to each well, and the plate was left to test for 5 min. Finally, the fluorescence intensity was read in a microplate reader (Fluostar^®®^ Omega, Ortenberg, Germany), with an excitation wavelength of 485 nm and an emission band of 590 nm. All fluorescence measurements were performed at room temperature [34].

### 2.8. Experimental Design and Statistical Analysis

A completely randomized experimental design was utilized, comprising each of the treatments (Np Q and Np QC) at five different concentrations (A, B, C, D, and E), as well as capsaicin in solution as control. We analyzed the differences between each of the concentrations within each treatment. The experimental data were analyzed by means of one-way analysis of variance (ANOVA) employing NCSS 97 software (NCSS, Inc., Kaysville, UT, USA) at a significance level of *p* = 0.05. The means analysis was carried out through a multivariate range Tukey test (Tukey post hoc test), at a 95% confidence interval. All of the results were presented with mean ± standard error.

## 3. Results and Discussion

### 3.1. Physical and Physicochemical Characterization of the Nanoparticles 

#### 3.1.1. Form, Size, and Zeta Potential

**Figure 1:**
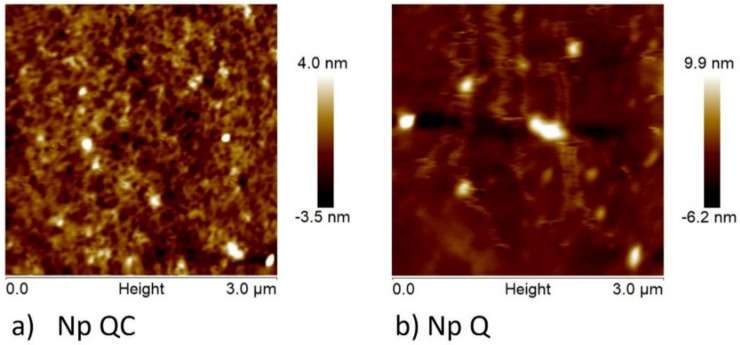
Atomic force microscopy (AFM) image of nanoparticles made of (**a**) capsaicin-loaded chitosan (Np QC) and (**b**) chitosan (Np Q).

In the nanoprecipitation method, the formation of nanoparticles is due to interphase turbulence between two non-equilibrated liquid phases, involving processes of flow, diffusion, and surface; thus, the size of the nanoparticles obtained is dependent on the conditions employed during this synthesis. The AFM images confirmed that the mean diameter of the nanoparticles made of chitosan–capsaicin (Np QC) and chitosan (Np Q) were of 116.6 ± 21 nm and 172.5 ± 28 nm, respectively (Figure 1). Both samples presented regular size distribution in addition to a uniform spherical shape. In the Np Q, an increase in the diameter of the particle can be observed with respect to the results found by DLS; the latter can be due to various factors, including the swelling of the chitosan layer that surrounds the individual particles and/or the aggregation of the particles while they were dispersed in water [35]. Additionally, it should be considered that these techniques (AFM and DLS) measure the diameters under different conditions, as proven in the present investigation. As shown in Table 2, the chitosan nanoparticles presented a mean diameter of 44.8 ± 20.6 nm, utilizing a chitosan concentration of 0.4 mg/mL and a 1.25 solvent/non-solvent proportion. This result differs from that of Chávez-Magdaleno et al. (2018) [29], who reported an average diameter of 341.2 ± 12.4 nm for nanoparticles made of chitosan (0.5 mg/mL) at a proportion of a 1:16 solvent/non-solvent relation, synthesized using the nanoprecipitation technique. On the other hand, in the synthesis of cellulose acetate nanoparticles (acetone/water/tetrahydrofuran), particle sizes of 60–140 nm were found upon varying the concentration of tetrahydrofuran from 10% to 30% in the non-solvent [36]. It has been documented that the effect on the organisms depends on the size of the nanoparticle; for example, nanoparticles measuring less than 300 nm can be absorbed by individual cells, while those that measure less than 70 nm can be absorbed by the nuclei of the cells [37]. Further, the size of the polymer nanoparticles plays an essential role in the properties and final applications of these materials [38].

The average value of the zeta potential of the chitosan nanoparticles was +25.6 ± 0.7 mV and that of the chitosan–capsaicin nanoparticles was +26.8 ± 6.1 mV (Table 2).t These positive values indicate the presence of amino groups on the surface of the particles. According to Harris et al. (2011) [39], the zeta potential positive values provide a mucoadhesive character and properties of absorption to the nanoparticles. Russo et al. (2014) [40] employed the ionotropic gelation method to prepare chitosan nanoparticles and Foscarnet (phosphonoformic acid salt), which is an antiviral agent, utilizing chitosan (0.5 mg/mL) at 0.5 mL/min and 400 rpm. The nanoparticles obtained presented diameters of 349 ± 6 nm, 195 ± 2 nm, and 208 ± 3 nm, containing Focsani concentrations of 8.8 mg/mL, 12 mg/mL, and 14 mg/mL, respectively. That investigation highlighted the effect of the concentration on the size of the nanoparticles; however, no effect was found in the zeta potential value upon varying the concentration of Foscarnet (+21.4 ± 0.5 mV, +22.4 ± 0.7 mV, and +21.7 ± 0.7 mV, respectively). On the other hand, an investigation conducted by Sullivan et al. (2018) [41] reported potential zeta values of +26 mV and a diameter of 186 nm for medium-molecular-weight chitosan nanoparticles synthesized by the ionotropic gelation technique. The zeta potential is an important parameter for analyzing the stability of nanoparticles in an aqueous medium [42,43].

#### 3.1.2. FT-IR and Nanoparticle Entrapment Capacity

**Figure 2:**
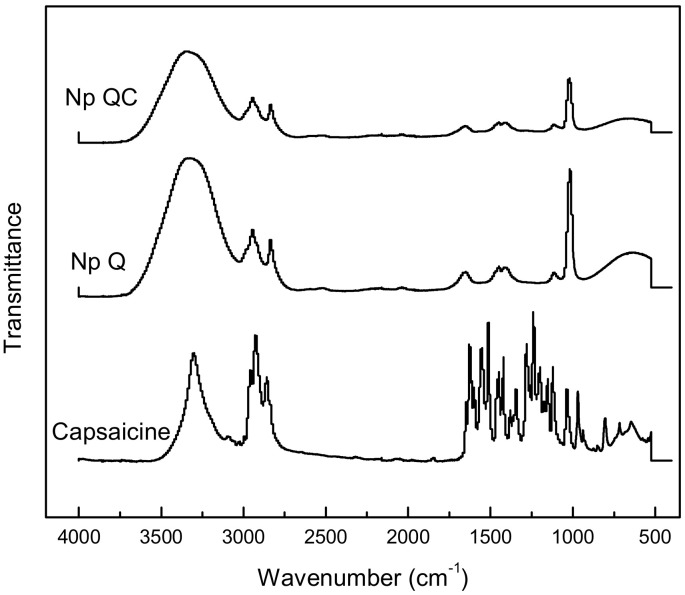
FTIR spectra of capsaicin, chitosan nanoparticles, and capsaicin loaded-chitosan nanoparticles.

The capsaicin molecule has proton donor atom N–H and O–H groups and proton acceptor atoms C=O and O–H groups, while the chitosan molecule includes three proton donor groups (N–H and two O–H). Hydrogen bonding can possibly be formed between these groups [11]. This means that inter- and intramolecular H bonds can be formed between the phenolic –OH or C=O groups of capsaicin and the –OH or NH_2_ groups of chitosan (Figure 2). A lesser intensity may be observed in the signal corresponding to the -NH_2_ of the Np QC with respect to the Np Q. In the capsaicin spectrum, there are signals near 1645 cm^−1^, which can be attributed to the C=O group present in the capsaicin. The latter is in agreement with an investigation carried out by our investigative group that evidenced the bond between the NH_2_ of the chitosan with the C=O group of the flavonoid (nobiletin) [25]. The inclusion of capsaicin in the Np QC was corroborated on quantifying the amount of capsaicin included in the nanoparticles. The latter was carried out by means of the quantification of capsaicin through ultra-HPLC, where it was proven that the percentage of capsaicin trapped in the chitosan-capsaicin nanoparticles was 81.78 ± 0.40%.

### 3.2. Toxicity of Nanoparticles 

#### 3.2.1. Acute Toxicity in *Artemia salina* Nauplii

**Figure 3:**
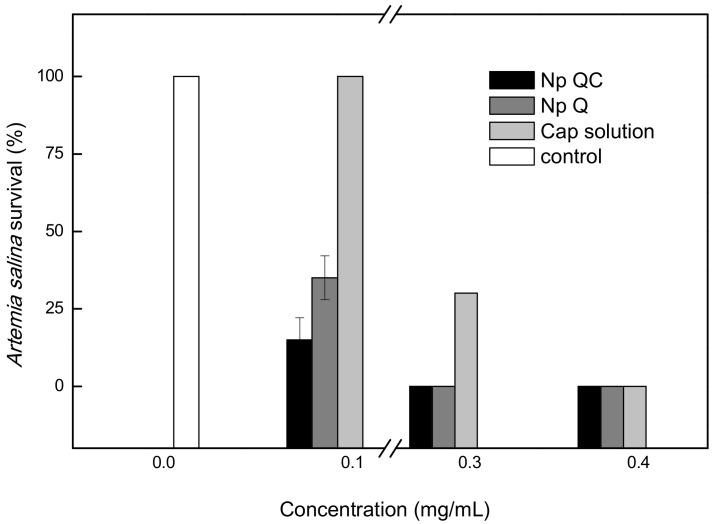
Acute toxicity of chitosan nanoparticles (Np Q), capsaicin loaded-chitosan nanoparticles (Np QC), and capsaicin solution on *A. saline*.

Figure 3 shows the results of the acute toxicity test. The effect of the treatment applied at different concentrations was observed. The 0.1 mg/mL concentration of the capsaicin control did not affect the survival of *A. salina*. In contrast, the Np Q treatment at that same concentration caused a diminution of 70% in survivors, while the treatment with Np QC diminished the survival of *A. salina* nauplii by 80%. At a concentration of 0.3 mg/mL of control capsaicin, there was a diminution of 73%, while at that same concentration, the Np Q and the Np QC did not present survivors. Finally, upon increasing the concentration to >0.4 mg/mL, the nauplii of *A. salina* did not survive in any of the treatments applied. 

#### 3.2.2. Acute Phytotoxicity in Lettuce Seeds (*Lactuca sativa*)

Table 3 shows the phytotoxicity results of the nanoparticles obtained. The control target of capsaicin at a concentration of 0.1 mg/mL allowed for the germination of 68% of the lettuce seeds, while the Np Q and the Np QC of the same concentration only permitted the germination of 12% of the treated seeds. Upon increasing the concentration of the Np Q and the Np QC to 0.2 and 0.3 mg/mL, seed germination was not observed. At these same concentrations, Np Q and Np QC were very toxic, presenting IG and IER indices with values of −1. Both the IGN and IER indices established toxicity values ranging from −1 to >0 under the following categories: index of 0 to −0.25, low toxicity; index of −0.25 to −0.5, moderate toxicity; index of −0.5 to −0.75, very toxic; and index of −0.75 to −1.0, very high toxicity. Index values > 0 indicate growth of the radicle or hormesis [44,45]. According to the United States Environmental Protection Agency (USEPA), nanoparticles can be reported with minimal toxicity if the tests in plants do not negatively affect the germination of the seeds [31]. It is known that the mechanisms of toxicity of nanoparticles can be related to the structure and chemical composition of the particle size and the surface area of contact [30]. The results obtained can help enhance the knowledge of toxicity in this type of nanomaterial. 

### 3.3. Antifungal Activity of the Nanoparticles of Chitosan (Np Q) and Chitosan-Capsaicin (Np QC) Nanoparticles on A. parasiticus

#### 3.3.1. Morphometric Analysis 

**Figure 4:**
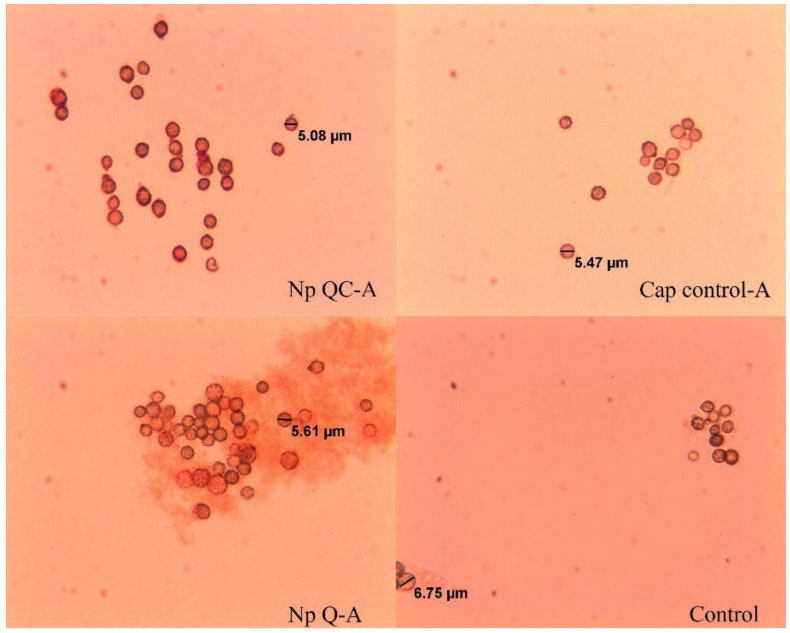
Spores of *A. parasiticus* grown in Czapek liquid media with an added Np Q-A, Np QC-A and capsaicin solution (Cap control-A), and control (liquid media), at 8 h after inoculation. The images were taken at 40×.

The average diameter of the spores of *A. parasiticus* is shown in Table 4. No differences were found (*p* > 0.05) in the average values of the diameter of the spores when they came into contact with the chitosan nanoparticles, the chitosan-capsaicin nanoparticles, and the capsaicin (control) at different concentrations. The diameter of the *A. parasiticus* spores did not exert an important effect, compared with the spores developed in the control medium, which indicates that the morphology of *A. parasiticus* was normal; this may be due to the fact that there are no cationic amino groups (NH_3_^+^) available that can interact with the membrane of the fungus (Cota-Arriola et al., 2017). The spores inoculated in the control media (capsaicin) and the non-treated spores (water control), exhibited continuity and a smooth and intact cell wall with little vesicular structure (Figure 4). However, in treatments such as Np Q-A and Np QC-A, damages were presented on the cell wall of the spores. These damages were characterized by an anomalous morphological structure, swelling, and heterogenous perimeter, which can be attributed to cell-wall changes in the pressure and tension of the fungus [46]. Similar results were published by Wang et al. (2015) [47], who, by means of scanning electronic microscopy, determined the effect of protonectin on the morphology of the fungal cells of *Candida glabrata*; according to that study, cells treated with protonectin had an abnormal morphological structure, with irregularities in the perimeter that indicated damage to the cell wall. The cells were surrounded by numerous vesicular structures. 

#### 3.3.2. Spore Viability 

**Figure 5:**
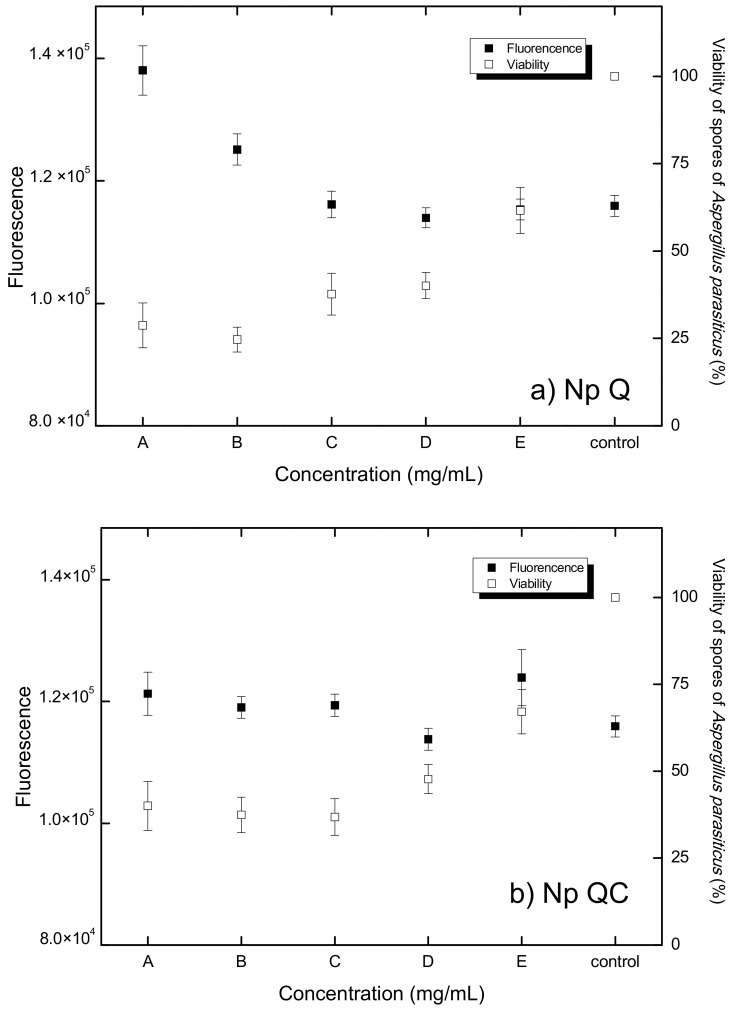

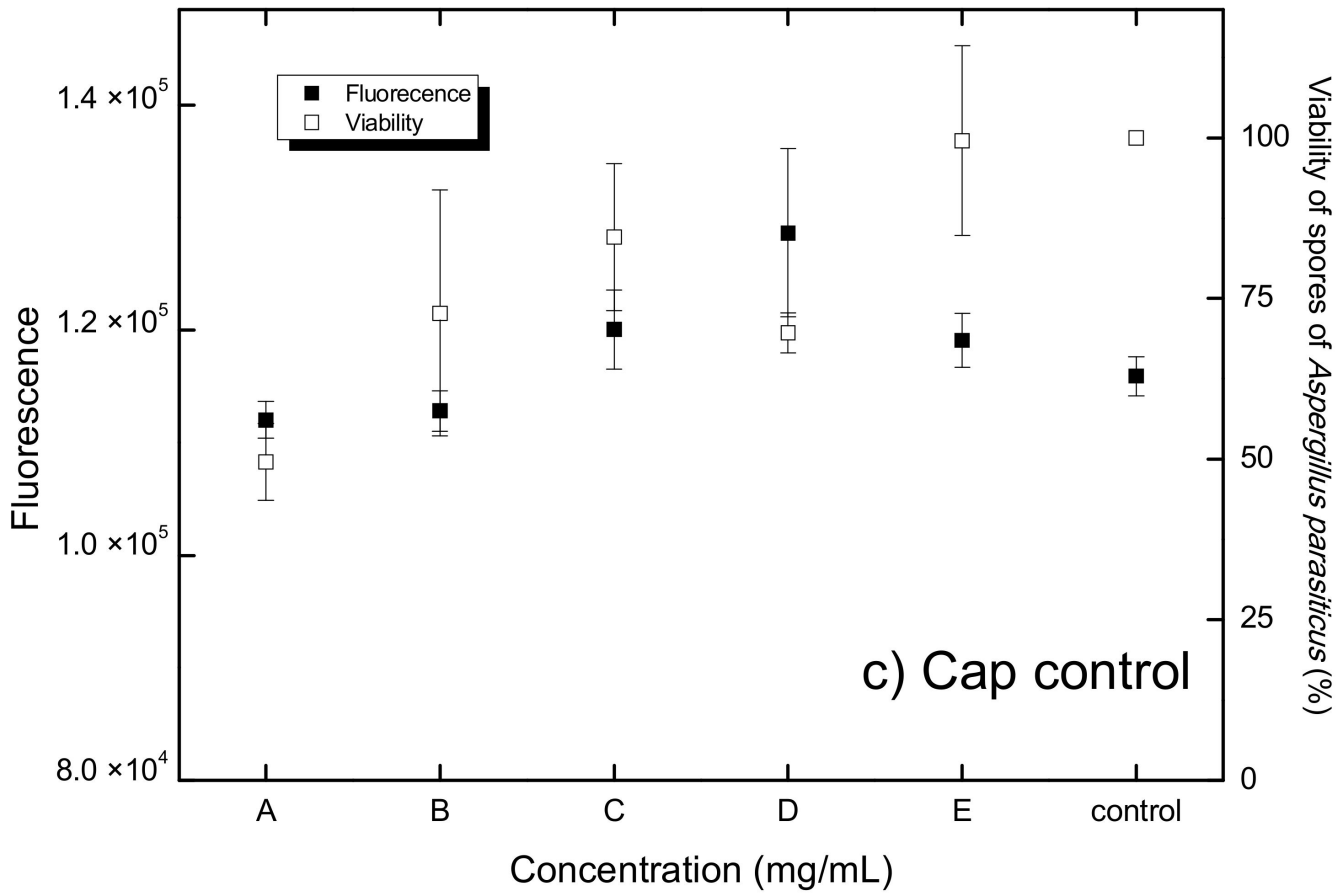
Relationship between ROS production (fluorescence) and viability of *A. parasiticus* spores exposed to: (**a**) chitosan nanoparticles (Np Q) at concentrations of A (0.4 mg/mL), B (0.3 mg/mL), C (0.2 mg/mL) D (0.1 mg/mL), and E (0.05 mg/mL); (**b**) chitosan-capsaicin nanoparticles (Np QC) at concentrations of A (chitosan 0.4 and capsaicin 0.75 mg/mL), B (chitosan 0.3 mg/mL and capsaicin 0.50 mg/mL), C (chitosan 0.2 mg/mL and capsaicin 0.25 mg/mL), D (chitosan 0.1 mg/mL and capsaicin 0.125 mg/mL), and E (chitosan 0.05 mg/mL and capsaicin 0.060 mg/mL); and (**c**) capsaicin solution (Cap control) at concentrations of A (0.75 mg/mL), B (0.50 mg/mL), C (0.25 mg/mL), D (0.125 mg/mL), and E (0.06 mg/mL).

Figure 5c shows the effect of the control solution of capsaicin at different concentrations (control) on the spores of *A. parasiticus*. The concentration of 751.9 µg/mL (Q-A) gave rise to a significant diminution (*p* < 0.05) in viability, which is in agreement with a previous report by Moreno et al. (2012) [6] on utilizing capsaicin at concentrations of 2, 1.6, 1.2, 0.8, and 0.4 mg/mL to inhibit the growth of *A. flavus*, using the agar well technique. Similarly, the results indicate that at concentrations of 0.2 mg/mL, 0.6 mg/mL, and 1.0 mg/mL, capsaicin exhibits inhibitory efficacy on *A. flavus* spores [48].

Chitosan-based nanosystems have aroused great interest in recent years, due to the versatility, biocompatibility, and biodegradability of chitosan. Diverse investigations have been carried out, especially for the propagation of mixed systems with improved properties, including those highlighted by flavonoids [25], essential oils [49], lysozyme [50], and benzol isothiocyanate [51]. The antimicrobial activity of chitosan has been explored in an extensive gamma of applications, from biomedicine to agriculture [52,53]. There is evidence of the effect of chitosan nanoparticles as an antioxidant, antibacterial, and antifungal agent [54,55,56]. In the present investigation, we verified that, as with the treatment based on chitosan nanoparticles (Np Q) and the treatment based on chitosan, capsaicin nanoparticles (Np QC) at different concentrations gave rise to the diminution of cell viability according to the manner in which the concentrations of the treatment increased (Figure 5a,b). Similar results were found by Saharan et al. (2013) [53], who obtained nanoparticles of chitosan and chitosan–saponin with an average diameter of 192.2 ± 2.5 nm and 373.9 ± 4.1 nm, respectively; in that study, chitosan nanoparticles had a greater antifungal effect, with respect to the chitosan-saponin nanoparticles, on *Macrophomina phaseolina* and *Rhizoctonia solani* phytopathogens, which was attributed to the size of the nanoparticles. This factor can be limiting in terms of penetration into certain parts of the fungi [37]. Another study that was in agreement was carried out by Divya et al. (2017) [54], who considered the effect of the diameter of chitosan nanoparticles in the viability of *Klebsiella pneumoniae*, upon applying different concentrations of chitosan nanoparticles (2 mg/mL, 3 mg/mL, and 4 mg/mL), with sizes of 21 nm, 120 nm, and 160 nm, respectively.

The antifungal effects of chitosan can vary, depending on the characteristics of the fungus. For example, with *A. parasiticus*, chitosan had a fungistatic effect, i.e., it was not capable of killing the fungus, nor did allow the fungus to continue to grow or to reproduce [57]. On the other hand, fungi that have chitosan in their cell wall possess greater resistance to antifungal effect, as in the case of *A. niger*, where 10% of the cellular membrane is made up of chitosan, which may explain the low sensitivity to this biopolymer [46]. In the present study, a minor spore viability was observed at higher concentrations of chitosan (Figure 5a), which we attribute to the previously known antifungal effects of chitosan as documented by some authors, including Ayala et al. (2014) [58], who utilized chitosan to eradicate a plague of banana leaf fungi. Chávez-Magdaleno et al. (2018) [29] indicated a similar behavior upon applying different concentrations of chitosan nanoparticles (40 µg/mL, 80 µg/mL, and 60 µg/mL) on *Colletotrichum gloeosporioides.* Similar results were reported in 2016 by Luque-Alcaraz et al. [15] who, upon analyzing the antifungal effect of chitosan nanoparticles on the viability of *A. parasiticus*, found a diminution of 25% when a concentration of 150 mg/mL of a chitosan-nanoparticle suspension was applied. Another factor to which this effect could be attributed is to the use of chitosan in the form of nanoparticles, because due to their small size, it is easier for them to penetrate the cell membrane with a greater effect on the cell [37], which can be attributed to its spherical form and its polycationic character, which interact with the negative charge of the surface of the microorganisms. The superficial area could allow its strong absorption into the microorganism’s surface, breaking through the membrane and causing the exit of intercellular compounds and, subsequently, cell death [54].

#### 3.3.3. Quantification of Reactive Oxygen Species (ROS)

The DCFHDA microplate method was employed to quantify free-radical-induced oxidative stress in cells. In this context, the results of Wang et al. (1999) [59] indicated that diverse free radicals can present a fluorescence concentration-dependent response, indicating the non-discriminant nature of DCFH. This behavior is depicted in Figure 5a, which shows the dependence of the fluorescence response upon varying the concentration of chitosan. In Figure 5c, it is shown that there is ROS production in the spores of *A. parasiticus* upon applying different concentrations of capsaicin in solution (control target). Differences in the production of ROS between the control spores and the different concentrations of (*p* > 0.05) capsaicin in solution were not found, which indicates that the solution of capsaicin does not produce oxidative stress in the spores of *A. parasiticus* at the different concentrations evaluated. However, it has been documented that other antifungal agents of natural origin, such as essential oils, exert an effect on the redox state of the spore, as in the case of dill essential oil, with which, at concentrations of 0.25, 0.5, 1.0, and 2.0 µL/mL on the spores, *A. flavus* induced high concentrations of ROS, caused by the mitochondrial dysfunction of the fungus. This same behavior was observed upon utilizing this same essential oil at concentrations of 0.156, 0.312, 0.625, and 1.25 µL/mL to induce oxidative stress in *Candida albicans* [60,61].

Figure 5b shows the production of ROS due to the effect of treatment based on chitosan–capsaicin (Np QC) nanoparticles. It was found that the spores of *A. parasiticus* did not reveal evidence of a production of ROS different from that observed in the spores developed in the control medium, suggesting that the treatment with Np QC nanoparticles does not cause oxidative stress in the spores. These results differ from those of an investigation that evaluated the effect of copper oxide nanoparticles and chitosan microparticles with concentrations of 0, 10, 30, and 90 mg/mL, in which this compound demonstrated that there is a correlation between these nanoparticles and the cytotoxicity generated by ROS, in that an alteration was observed in the DNA, in the protein, and in the cellular membrane after the induction of these nanoparticles [62]. Other results have shown the effect of chitosan nanoparticles loaded with ethanolic extracts of *Gymnemate Sylvester* and *Cinnamomum zeylanicum* at concentrations of 100 g/mL and 200 g/mL, respectively. In this assay, it was observed that there was an increase in the production of ROS through the effect of the chitosan–ethanolic extract nanoparticles and that there was a diminution in the cellular proliferation rate, or even cell death (in SiHa cervical cancer human cell lines) through the apoptotic or necrotic routes [63].

Figure 5 shows the oxidative stress response of the *A. parasiticus* spores to the different concentrations of the Np Q treatment. There were no significant differences in the response of the spores to the concentrations of Np Q (B, C, D, and E) nanoparticles, with respect to the control spores. In the meantime, the concentration of Q-A (600 µg/mL) presented a significant difference (*p* > 0.05) with respect to the control, i.e., a greater production of ROS with respect to the control. Chitosan nanoparticles at concentrations of Q-E, Q-D, Q-C, and Q-B induced oxidative stress, as did the control treatment in the spores of *A. parasiticus*. This can be due to the fact that that the concentrations are low in terms of causing a change in the spore’s redox state. On the other hand, a Np Q-A concentration with 600 µg/mL can give rise to stress in the fungus. This same effect was observed upon applying chitosan nanoparticles to inhibit the growth of *Pyricularia grisea* in finger millet plants; in such an assay, the plants treated with nanoparticles had a greater accumulation of ROS and peroxidases in comparison with the control plants [55]. In addition to an ability to generate a certain resistance, fungi possess a system of ROS detoxification, which includes superoxide dismutase, catalases, peroxidases, glutathione peroxidases, and peroxiredoxins, and which ensures a change of ROS in order to maintain redox homeostasis. This detoxification system permits the cell to protect itself from the free radicals that could affect its structure or functioning [64].

Oxidative stress can occur due to the inequality between the generation and elimination of reactive oxygen species (ROS), in such a way that the existence of high concentrations of ROS inflict indiscriminate damage on the biomolecules, with an imbalance that could cause cell death. As evidenced in these results, there can be a clear indication of the relationship between the production of ROS and the cellular viability of *A. parasiticus*. As shown in Figure 5a, there is an inverse relation between the production of ROS and cell viability, in terms of the effects occasioned by the Np A-Q treatment at the greatest concentration tested. With an increased production of ROS, there is lower spore viability of *A. parasiticus*. This same effect has been found by Hamdi et al. (2018) [65], who concluded that epoxiconazole is capable of inducing a diminution in the cell viability of HCT116 cells, predominantly attributable to the generation of ROS that, in turn, induced mitochondrial dysfunction and fragmentation of the DNA, resulting in cell death. Their hypothesis was confirmed, because cell death induced by epoxiconazole was clearly diminished upon treating the cells with an antioxidant agent. 

Similarly, a published study carried out with *C. albicans* yielded results that indicated the inarguable role of the production of ROS in the death of fungal cells, because the authors of that study discovered that the accumulation of ROS in endoplasmic-reticulum fungal cells promotes the oxidation of ERO1, an important source in the production of ROS [66]. The results of that study revealed that the oxidation of ERO1 in the endoplasmic reticulum contributes to cell-wall stress, which is related to the production of ROS and, consequently, with the inhibition of cell viability. The antifungal peptides also presented similar behavior in terms of the production of ROS. Wang et al. (2015) [47] investigated the antifungal activity of protonectin, an antimicrobial cationic peptide. The results of their study showed that the peptide was effective against *C. albicans* and *C. glabrata*, in that it induced an increase in the production of ROS and a decrease in viability, depending on the concentration of the peptide that was utilized.

## 4. Conclusions

Particles in the nanometric range with positive ζ-potential were obtained by using the nanoprecipitation technique. The experimental evidence suggests that the Np QC reduce the viability of *A. parasiticus* conidia by inducing oxidative stress. In terms of the ability to reduce the germination of lettuce seeds and the survival of *A. salina*, Np QC presented a lower toxicological effect in comparison with the Np Q nanoparticles. The results suggest that the incorporation of bioactive compounds, such as capsaicin into chitosan nanoparticles, is a strategy that permits reducing the toxicity associated with nanostructured materials. The antifungal activity of these nanometric systems could become an environmentally safe alternative to the use of synthetic fungicides.

## Figures and Tables

**Table 1:** Nomenclature used to describe the concentration of both chitosan nanoparticles and chitosan–capsaicin nanoparticles applied in *A. parasiticus* spores for toxicity and antimicrobial activity.

	Treatment	Chitosan Concentration (mg/mL)	Capsaicin Concentration (mg/mL)
Chitosan nanoparticles			
	Np Q-A	0.4	-
	Np Q-B	0.3	-
	Np Q-C	0.2	-
	Np Q-D	0.1	-
	Np Q-E	0.05	-
Chitosan–capsaicine nanoparticles			
	Np QC-A	0.4	0.75
	Np QC-B	0.3	0.50
	Np QC-C	0.2	0.25
	Np QC-D	0.1	0.125
	Np QC-E	0.05	0.06
Capsaicin control (capsaicin in solution)			
	Cap control-A	-	0.75
	Cap control-B	-	0.50
	Cap control-C	-	0.25
	Cap control-D	-	0.125
	Cap control-E	-	0.06
Control			
	Control	0	0

**Table 2:** Diameter and zeta potential of chitosan nanoparticles (Np Q) and chitosan–capsaicin nanoparticles (Np QC).

	Diameter (nm)	Zeta Potential (mV)
Np Q	44.8 ± 20.6 ᵃ	+25.6 ± 0.71 ᵃ
Np QC	111.1 ± 14.1 ᵇ	+26.8 ± 6.14 ᵃ

Data, followed by standard errors, are means of at least three experiments. Treatment means were separated using the Tukey test (*p* < 0.05). ^a,b^ Different letters in superscript indicate significant differences in the column (*p* < 0.05).

**Table 3:** Inhibition of seed germination and elongation of radicle and hypocotyl of *L. sativa* seeds exposed to Np Q and Np QC treatments, and capsaicin in solution for 120 h of storage at 24 ± 0.1 °C.

Treatment	IER (%) ^a^	IGN (%) ^b^	RSG (%) ^c^	RRE (%) ^d^	GI ^e^
Chitosan nanoparticles					
Np Q-A (0.4 mg/mL)	−1	−1	0	0	0
Np Q-B (0.3 mg/mL)	−1	−1	0	0	0
Np Q-C (0.2 mg/mL)	−1	−0.88	12.00	0	0
Chitosan/Capsaicine nanoparticles					
Np QC-A (0.4 mg/mL)	−1	−1	0	0	0
Np QC-B (0.3 mg/mL)	−1	−1	0	0	0
Np QC- C (0.2 mg/mL)	−1	−0.88	12.00	0	0
Capsaicine in solution					
Cap control-A (0.4 mg/mL)	−1	−1	0	0	0
Cap control- B (0.3 mg/mL)	−1	−0.76	24.00	0	0
Cap control-C (0.2 mg/mL)	−0.5084	−0.32	68.00	49.15	33.44
Control (H_2_0)	0	0	100	100	100

^a^ IER (%) = normalized residual elongation of the root of the germinated seeds per treatment. ^b^ IGN (%) = normalized residual percentage of germinated seeds after the experiment. ^c^ RSG (%) = relative seed germination. ^d^ RRE (%) = relative radicle elongation. ^e^ GI (%) = germination index. Mean values, *n* = 3.

**Table 4:** Spore diameter of *A. parasiticus* exposed to Np Q and Np QC nanoparticles and capsaicin in solution. The figures indicate significant difference with respect the water control at *p* ≤ 0.05, according to the Tukey test.

Treatment	Diameter (µm)	Treatment	Diameter (µm)	Cap Control (Capsaicin Solution)	Diameter (µm)
Np Q-A	5.30 ± 0.5793 ^a^	Np QC-A	5.34 ± 0.5481 ^a^	C-A	5.07 ± 0.8492 ^a^
Np Q-B	5.18 ± 0.7545 ^a^	Np QC-B	5.50 ± 0.6439 ^a^	C-B	5.39 ± 0.7043 ^a^
Np Q-C	5.83 ± 0.6640 ^a^	Np QC-C	6.14 ± 0.8612 ^a^	C-C	5.55 ± 0.8425 ^a^
Np Q-D	6.24 ± 0.7329 ^a^	Np QC-D	5.23 ± 0.6117 ^a^	C-D	5.41 ± 0.7497 ^a^
Np Q-E	5.85 ± 0.9189 ^a^	Np QC-E	5.94 ± 1.0180 ^a^	C-E	5.51 ± 0.8526 ^a^
Control	5.35 ± 0.8363 ^a^	Control	5.35 ± 0.8363 ^a^	Control	5.35 ± 0.8363 ^a^

The data are the mean and the standard error, *n* = 50. ^a^ Different superscript letters for each treatment show statistical differences (*p* ≤ 0.05).

## Data Availability

Not applicable.
